# Supplementary material for: Content-rich biological network constructed by mining PubMed abstracts
Source: BMC Bioinformatics. 2004 Oct 8;5:147. doi: 10.1186/1471-2105-5-147 (PMC528731; doi:10.1186/1471-2105-5-147)
Supplement: Additional File 2 — The original results of the above study (non-essential files are deleted to keep the file size under the limit set by BMC bioinformatics). [file 1471-2105-5-147-S2.bz2 › chilibotAdditionalFile2/dip20/396ID10775655E9950/html/EGFR_TGFA.html]

 


 **EGFR** and **TGFA** 
  
Found 1071 abstracts in PubMed, retrieved 20.  
 

 What does Google say? 
 PDF only 
| .edu only 

---

**Interactive relationship** (e.g. stimulation, inhibition, etc)

**Inhibitory relationship**- Our results demonstrate that E4T and  **TGFa**  provoke decreased Cbl recruitment,  **EGFR**  ubiquitination and  **EGFR**  degradation compared to EGF.  Ref: 12829707 J Biol Chem< MedlineTA>J Biol Chem,
- We conclude that the enhanced  **EGFR**  recycling that has been observed in HER 14 cells following  **TGFa**  or E4T stimulation correlates with decreased  **EGFR**  ubiquitination and  **EGFR**  degradation,.  Ref: 12829707 J Biol Chem< MedlineTA>J Biol Chem,
- Co treating PC 3 cells with quercetin significantly attenuated EGF and TGF alpha  [ **TGFA** ]  induced growth and phosphorylation of ErbB  [ **EGFR** ]  2, ErbB  [ **EGFR** ]  3, c Raf, MAPK kinase 1 2 MEK1 2, MAPK, Elk 1 and Akt 1.  Ref: 12888923 Int J Oncol, 2003
**Neutral relationship**- In contrast, the amount of TGF alpha  [ **TGFA** ]  bound to  **EGFR**  is found to be higher in the gastric mucosa of aged than in young rats.  Ref: 12772780 Dig Dis Sci, 2003
- Our simulations predict that when  **EGFR**  is activated with TGF alpha  [ **TGFA** ] , receptor activation is biased toward the cell surface whereas EGF produces a signaling bias toward the endosomal compartment.  Ref: 12885624 Biophys J, 2003
- The epidermal growth factor receptor  [ **EGFR** ]  is a transmembrane glycoprotein that mediates the cellular responses to epidermal growth factor EGF and transforming growth factor alpha  [ **TGFA** ]  TGF alpha  [ **TGFA** ] .  Ref: 12731890 Biochemistry, 2003
- The TGF alpha  [ **TGFA** ]  induction of AP 1 transcriptional activity in gastric mucosal cells from aged rats to could be totally abrogated by either PD153035, a specific inhibitor of  **EGFR**  tyrosine kinase, or PD98059, a specific inhibitor of MEKs, but not by Wortmannin, which inhibits PI 3 kinase.  Ref: 12672650 Am J Physiol Gastrointest Liver Physiol< MedlineTA>Am J Physiol Gastrointest Liver Physiol, 2003

**Non-interactive relationship** (e.g. studied together, co-existance, homology, etc.)

- Induction of DNA synthesis in primary mouse hepatocytes is associated with nuclear pro transforming growth factor alpha  [ **TGFA** ]  and erbb 1  [ **EGFR** ]  and is independent of c jun.  Ref: 12771026 Carcinogenesis, 2003
- Immunohistochemical procedures were performed to detect the following protein antigens CD99, interleukin IL 1alpha, IL 6, transforming growth factor TGF alpha  [ **TGFA** ] , conventional c protein kinase C cPKC alpha, cPKC betaII, phosphorylated p PKC alpha betaII, c kit CD117, platelet derived growth factor receptor PDGFR alpha, PDGFR beta, epidermal growth factor receptor  [ **EGFR** ]   **EGFR** , human epidermal growth factor receptor  [ **EGFR** ]  HER 2 neu, cathepsin D, angiotensin converting enzyme ACE, angiotensin II type 1 AT1 receptor, p21ras, the alpha subunit of farnesyl and geranylgeranyl transferase FTalpha GGTalpha, phospho p c Jun N terminal kinase p JNK, p p38 mitogen activated protein kinase MAPK, cyclin D1, c Jun, Ki 67, bcl 2, TGF beta1 latency associated peptide LAP, TGF betaRII, and cyclooxygenase COX 2.  Ref: 12817616 Ann Clin Lab Sci< MedlineTA>Ann Clin Lab Sci, 2003
- Detection of transforming growth factor alpha  [ **TGFA** ]  and epidermal growth factor receptor  [ **EGFR** ]  mRNA and immunohistochemical localization of their proteins in the ovine uterus during the early implantation period.  Ref: 12814185 Histochem J< MedlineTA>Histochem J, 2003
- RT PCR transcripts of  **EGFR**  ligands EGF, TGF alpha  [ **TGFA** ] , HB EGF, and amphiregulin were detected in both carcinomas and normal mucosa, indicating that autocrine growth stimulation of colorectal carcinomas is mediated by coexpression of EGF receptor ligands and upregulation of EGF receptors.  Ref: 12790326 Recent Results Cancer Res, 2003
- Here we evaluated  **EGFR**  activation, Cbl recruitment,  **EGFR**  ubiquitination and degradation in response to EGF,  **TGFa**  and E4T.  Ref: 12829707 J Biol Chem< MedlineTA>J Biol Chem, 2003
- To determine the utility of this approach, we simulated the differential activation of the  **EGFR**  by two of its ligands, epidermal growth factor EGF and transforming growth factor alpha  [ **TGFA** ]  TGF alpha  [ **TGFA** ] .  Ref: 12885624 Biophys J, 2003
- We hypothesize that the expression of epidermal growth factor receptor  [ **EGFR** ]   **EGFR** , epidermal growth factor EGF, and transforming growth factor alpha  [ **TGFA** ]  TGFalpha in GCT have a role in the development of PEH overlying some GCT.  Ref: 12715330 Ann Diagn Pathol, 2003
- CONCLUSION The expression levels of SF HGF and TGF alpha  [ **TGFA** ]  as well as their respective receptors, MET and  **EGFR** , are known to correlate with glioma malignancy grade.  Ref: 12762884 Neurosurgery, 2003
- Similarly,  **EGFR**  TK inhibition by ZD1839 reversed the invasive phenotype promoted by the constitutively activated form of the  **EGFR**  EGFRvIII and the  **EGFR**  agonists transforming growth factor alpha  [ **TGFA** ]  TGFalpha, amphiregulin and EGF.  Ref: 12881705 Oncogene, 2003
- The primary objective of this investigation is to determine the role of signaling pathways initiated by  **EGFR**  in regulating the TGF alpha  [ **TGFA** ]  induced activation of AP 1 in the gastric mucosa in aged rats.  Ref: 12672650 Am J Physiol Gastrointest Liver Physiol< MedlineTA>Am J Physiol Gastrointest Liver Physiol, 2003
- Transforming growth factor alpha  [ **TGFA** ]  TGF alpha  [ **TGFA** ] , a ligand of the epidermal growth factor receptor  [ **EGFR** ] , reduces the infarct size after focal cerebral ischemia in rat but the molecular basis underlying the protection are unknown.  Ref: 12771152 J Biol Chem< MedlineTA>J Biol Chem, 2003
- So, this study confirms the development of human olfactory mucosa and shows epidermal growth factor receptor  [ **EGFR** ] , transforming growth factor alpha  [ **TGFA** ]  and nerve growth factor beta proteins are reliable markers for developing olfactory epithelium.  Ref: 12739843 Growth Dev Aging< MedlineTA>Growth Dev Aging, 2003
- Inhibition of ErbB  [ **EGFR** ]  2 and ErbB  [ **EGFR** ]  3 expression by quercetin prevents transforming growth factor alpha  [ **TGFA** ]  TGF alpha  [ **TGFA** ]  and epidermal growth factor EGF induced human PC 3 prostate cancer cell proliferation.  Ref: 12888923 Int J Oncol, 2003
- In this study, standard immunohistochemical methods were used to detect the presence of epidermal growth factor receptor  [ **EGFR** ] , transforming growth factor alpha  [ **TGFA** ]  and nerve growth factor beta in the human olfactory epithelium of different developmental stages 7 to 30 weeks in formalin fixed tissue specimens.  Ref: 12739843 Growth Dev Aging< MedlineTA>Growth Dev Aging, 2003
